# Symm-CGNN: Symmetry-Information-Enhanced Crystal Graph Neural Network for High-Symmetry Point Band Gap Prediction

**DOI:** 10.3390/nano16140871

**Published:** 2026-07-15

**Authors:** Qihang Xu, Jian Wu, Xiuying Zhang, Sicong Zhu

**Affiliations:** 1 The State Key Laboratory for Refractories and Metallurgy, Hubei Province Key Laboratory of Systems Science in Metallurgical Process, School of Physics and Mechanics, Wuhan University of Science and Technology, Wuhan 430081, China; xqh0213@wust.edu.cn; 2 College of Advanced Interdisciplinary Studies, National University of Defense Technology, Changsha 410073, China; wujian15@nudt.edu.cn; 3 School of Physics and Mechanics, Wuhan University of Technology, Wuhan 430070, China; xyzhang25@whut.edu.cn

**Keywords:** Symm-CGNN, high-symmetry point band gap, global symmetry information, CGCNN, anisotropic optoelectronic properties

## Abstract

Accurately characterizing the anisotropic optoelectronic properties of crystals requires determining the band gaps at specific high-symmetry points in the Brillouin zone. Relying solely on the minimum band gap is insufficient. However, although Graph Neural Networks (GNNs) offer rapid property predictions, conventional models remain trapped in a local real-space paradigm, lacking the global symmetry information necessary to differentiate these high-symmetry energy states. To address this problem, we propose the Symmetry-Information-Enhanced Crystal Graph Neural Network (Symm-CGNN). It explicitly incorporates local atomic environments with global symmetry information, including space groups, crystal systems, material density and lattice constants. The evaluation is performed on a comprehensive dataset including 3D (Materials Project) and 2D (2DMatpedia). The results demonstrate that Symm-CGNN achieves an 18% reduction in Mean Absolute Error (MAE) for high-symmetry band gap prediction compared to the baseline Crystal Graph Convolutional Neural Networks (CGCNN). This approach bridges the representational gap between local atomic coordination and macroscopic symmetry. Consequently, it provides a robust and efficient machine-learning paradigm for the high-throughput screening of materials with anisotropic optoelectronic properties.

## 1. Introduction

**Figure 1:**
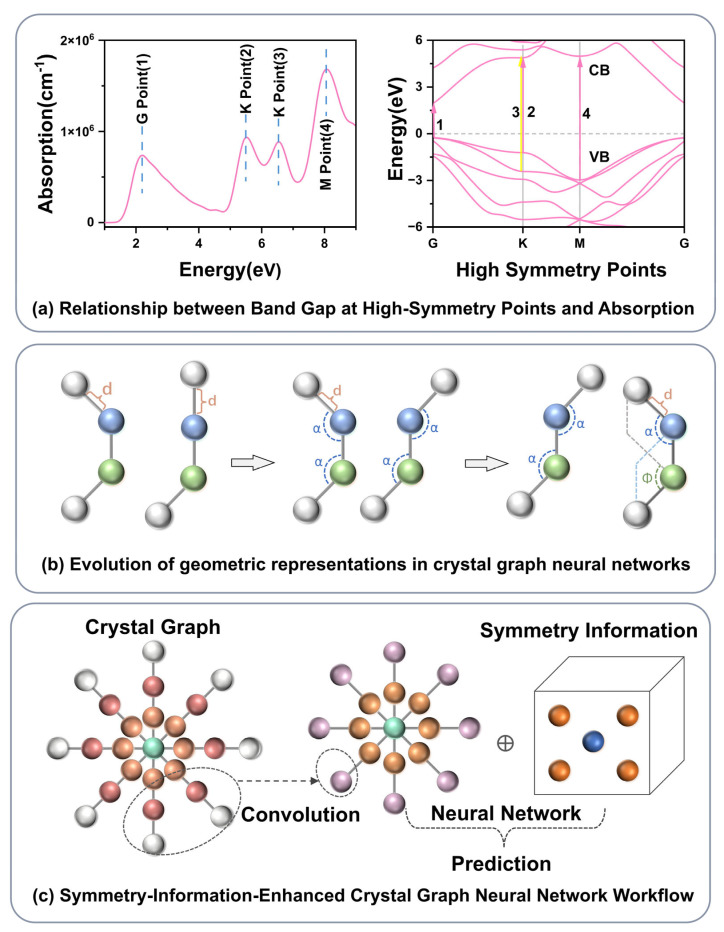
Physical motivation for predicting high-symmetry band gaps and the proposed deep learning architecture. (**a**) Schematic illustration bridging the macroscopic optical absorption spectrum (**left**) and the electronic band structure (**right**). The distinct absorption peaks directly correspond to specific vertical interband transitions (denoted as 1–4) occurring at various high-symmetry k-points (K, M, G). (**b**) The evolution and inherent limitations of conventional local deep learning models. (**c**) The workflow of the proposed Symm-CGNN.

The minimum band gap is a key parameter for evaluating the optoelectronic properties of semiconductors. However, this single scalar is insufficient to fully characterize complex photoexcitation processes [1,2,3,4,5,6,7,8,9,10,11,12,13,14]. It is also necessary to investigate band-edge renormalization phenomena, which typically arise from band gap differences at various high-symmetry points in the Brillouin zone [15,16,17,18]. The optical absorption profile of a material is governed by a series of vertical interband transitions at specific k-points. Instead of being defined solely by the minimum band gap, distinct absorption peaks correspond to local energy gaps at specific high-symmetry points, such as the G, K, and M points (Figure 1a). Therefore, determining the band gaps at these high-symmetry points is necessary for accurately mapping the optical absorption spectrum.

Although standard first-principles calculations provide accurate high-symmetry band gaps [19,20,21], their computational cost limits their application in high-throughput screening of large-scale materials databases [22,23,24,25,26]. To address this limitation, deep learning techniques have emerged as efficient approaches for material property prediction. In general, such models characterize the geometric features of crystals based on local structures. Model architectures have evolved to capture increasingly complex local structures, moving from bond lengths (e.g., CGCNN, SchNet) to bond angles (e.g., MEGNet) and dihedral angles (e.g., SphereNet) (Figure 1b). Despite these advancements, they remain confined to local real-space representations. Conventional models often fail to accurately predict high-symmetry band gaps due to the absence of global symmetry information [27,28,29,30,31,32,33]. Consequently, these local models exhibit limitations in distinguishing electronic state differences between various high-symmetry points, which restricts their prediction accuracy [34,35,36,37].

In this work, we propose a deep learning framework, Symm-CGNN, which incorporates global symmetry information. Built upon the CGCNN architecture, this model retains local chemical environment modeling while integrating global symmetry constraints to provide a representation of electronic structures [38,39,40]. To integrate these global structural features, specific encoding strategies are employed [41]. Raw crystal variables are mapped into continuous vector representations to preserve essential structural relationships [42]. As shown in the workflow (Figure 1c), Symm-CGNN extracts local structural features through graph convolutions and encodes macroscopic crystal properties as symmetry information. By combining these local and global features, the model accounts for overall crystal symmetry, leading to improved prediction accuracy of band gaps at specific high-symmetry points. Notably, this framework achieves a 26% reduction in MAE for high-symmetry band gap prediction compared to the baseline CGCNN.

## 2. Model Architecture

**Figure 2:**
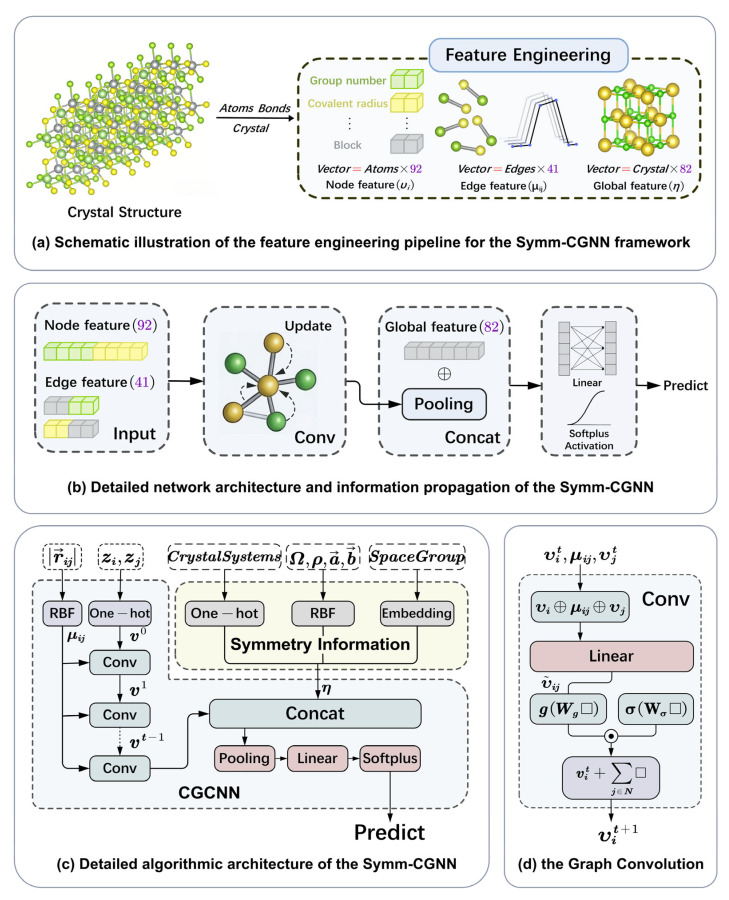
Comprehensive architecture and algorithmic workflow of the Symm-CGNN framework. (**a**) Schematic illustration of the feature engineering pipeline, mapping the raw crystal structure into explicit node (atom), edge (bond), and global (macroscopic) representations. (**b**) network architecture and information propagation, highlighting the extraction of local atomic environments via graph convolutions and the subsequent late-fusion of global features. (**c**) Detailed algorithmic schematic. Continuous spatial variables (e.g., distance, volume) and discrete crystallographic properties (e.g., space group, crystal system) are encoded via Gaussian radial basis functions (RBF) and embedding/one-hot layers to form the global context (η), which is then concatenated with the pooled local graph features. (**d**) Inner workings of the graph convolution block, demonstrating the local message-passing mechanism updated via gated linear operations.

Based on the CGCNN architecture, Symm-CGNN constructs graph representations directly from Crystallographic Information Files (CIFs). In this topology, atoms are represented as nodes encoded with elemental properties (Table 1), and chemical bonds within a defined cutoff radius are represented as edges encoded with interatomic distances (Figure 2a).

To supplement the local graph, macroscopic structural properties, including crystal volume (Ω), crystal density (ρ), lattice constants ($\vec{a},\vec{b}$), crystal systems, and space groups (Table 2), are extracted as global features (η). During information propagation (Figure 2b), node and edge features are processed through multiple graph convolutional layers to iteratively aggregate local structural information. A pooling operation then aggregates the updated node features into a graph-level representation, which is concatenated with the global features (η). Finally, this combined representation passes through linear layers to predict the band gap.

The feature encoding workflow processes both discrete and continuous variables (Figure 2c). Discrete categorical features (e.g., crystal systems and space groups) are represented using one-hot encoding and learnable embedding layers. Conversely, Gaussian radial basis functions (RBFs) are used to expand continuous variables. Inspired by PhysNet [42], RBFs are used to map both interatomic distances and continuous macroscopic features (e.g., volume and density) into high-dimensional vector representations, as expressed in Equation (1):

(1) $$\mu_{ij}=\exp\left(-\beta_{k}\cdot {\left(\exp\left(-\left|r_{ij}\right|\right)-\nu_{k}\right)}^{2}\right)$$

where $r_{ij}$ denotes the distance between atom i and atom j, while $\beta_{k}$ and $v_{k}$ are learnable parameters of the k-th Gaussian basis function, used to adjust the center position and distribution width of the basis functions. Through the above formulation, interatomic distances are transformed into vectors of appropriate dimensionality, serving as edge feature vectors $\mu_{ij}$.

Subsequently, through multiple convolutional layers, each node aggregates information from itself and its neighboring nodes, thereby updating the representations of all nodes in the graph. The main formulations are given in Equations (2) and (3):

(2) $$v_{i}^{t+1}=v_{i}^{t}+\sum_{j,k}\sigma \left(z_{i,j}^{t}W_{f}^{t}+b_{f}^{t}\right)⊙g\left(z_{i,j}^{t}W_{f}^{t}+b_{f}^{t}\right)$$

(3) $$z_{ij}^{t}=v_{i}^{t}⊕u_{ij}⊕v_{j}^{t}$$

where ⊙ denotes element-wise multiplication, $W_{f}^{t}$ and $b_{f}^{t}$ represent the weight matrix and bias vector of the t-th convolutional layer, respectively, σ denotes the Sigmoid function, g denotes the Softplus function, and ⊕ represents the concatenation of node features and edge features. Figure 2d illustrates the detailed process by which the convolutional layers aggregate feature information from each node and its neighboring nodes.

After multiple convolutional layers, a pooling layer is applied to generate the overall crystal feature vector $v_{c}$. For simplicity, normalized summation is adopted as the pooling function, as shown in Equation (4).

(4) $$v_{c}=SUM\left(v_{0}^{t},v_{1}^{t},v_{2}^{t}\cdots v_{N}^{t}\right)$$

Finally, the overall feature vector $v_{c}$ and the global feature vector η are combined through hidden layers to capture the complex mapping between crystal structures and their properties. An output layer is then connected to the hidden layers to predict the target properties.

## 3. Results

**Figure 3:**
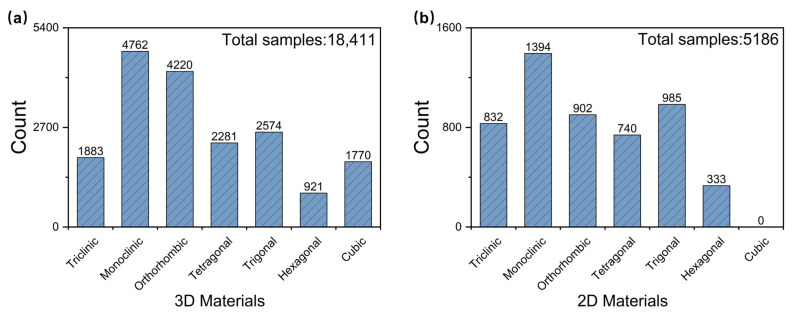
Crystal system distribution of materials in the dataset across different dimensionalities. (**a**) Sample counts of the seven major crystal systems for the 3D materials. (**b**) Sample counts of the crystal systems for the 2D materials.

**Figure A1:**
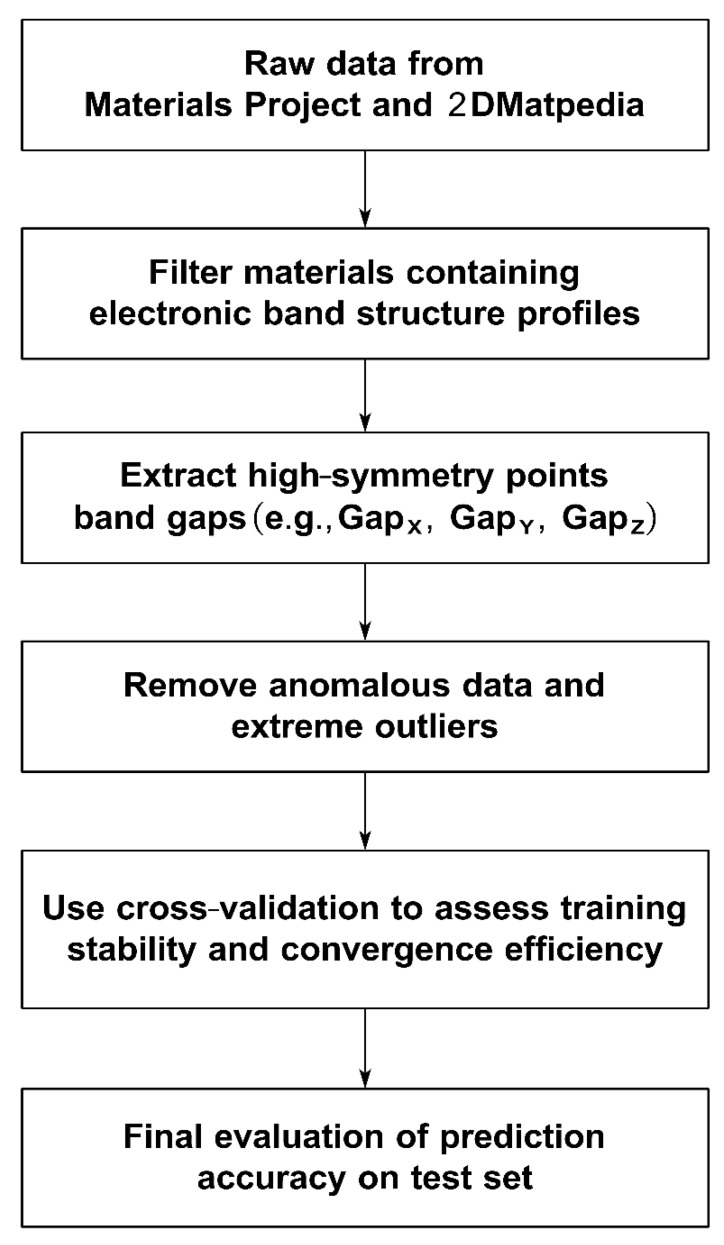
Comprehensive workflow of the data curation and model evaluation. The process begins with raw data from the Materials Project and 2DMatpedia databases. Subsequent data screening involves filtering materials with complete electronic band structures, extracting specific high-symmetry band gaps, and rigorously removing extreme outliers. The curated dataset is then subjected to a dual-phase assessment: cross-validation to verify training stability and convergence efficiency, followed by a final evaluation on an independent test set to ascertain the ultimate prediction accuracy.

**Figure A2:**
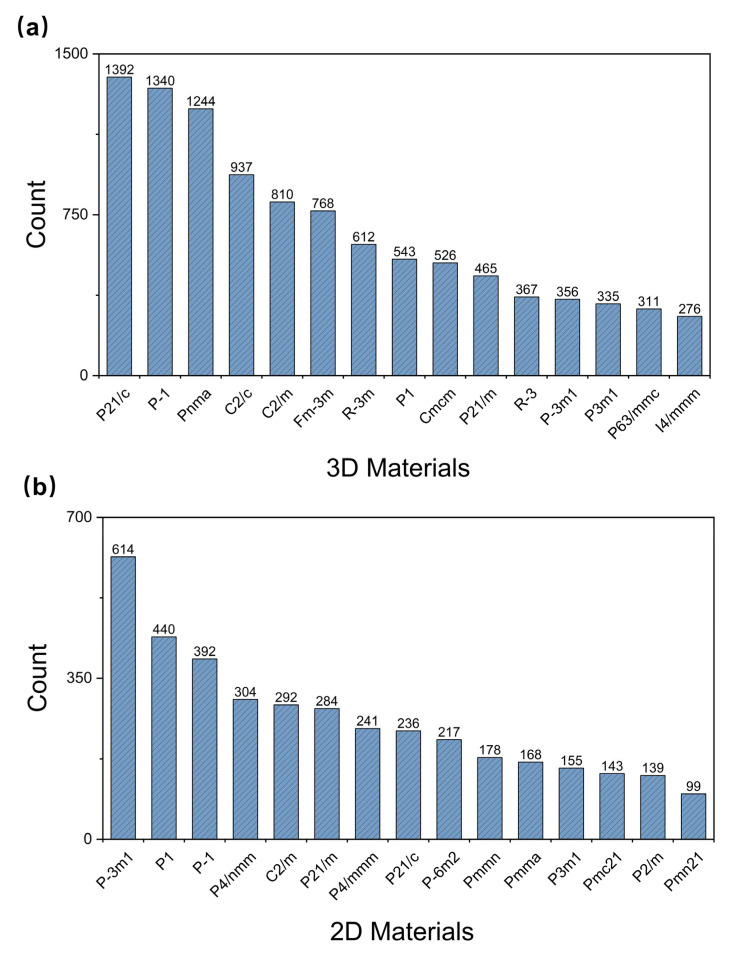
Distribution of the top 15 most frequent space groups in the dataset. (**a**) Space group frequency distribution for 3D materials; (**b**) Space group frequency distribution for 2D materials.

**Figure A3:**
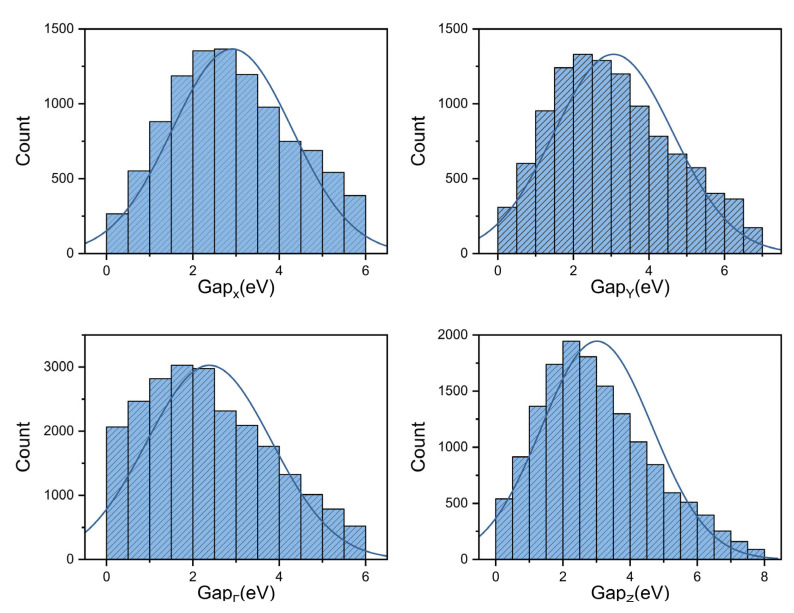
Statistical distribution of target high-symmetry band gaps in the curated dataset. The histograms display the frequency distributions of local energy gaps extracted at various critical high-symmetry points in the Brillouin zone. The solid blue curves indicate the overlaid continuous probability density estimations.

The model was trained and evaluated on a combined dataset constructed from the Materials Project (MP) [43] (comprising 3D crystal structures) and the 2DMatPedia (2DMat) [44] (comprising 2D materials) databases. Training on a unified dataset that includes both three-dimensional and two-dimensional materials not only enhances the diversity of the data distribution but also improves the model’s ability to represent materials with different structural dimensionalities. The dataset spans seven major crystal systems, with the overall distribution pattern remaining consistent across both the 3D and 2D material subsets. This distribution provides diverse and representative structures in terms of both structural dimensionality and crystal symmetry, offering a wide range of training examples for the model to capture structural variations (Figure 3). The frequency distribution of the dominant space groups is detailed in Figure A2. In the selection of prediction targets, we analyzed the band structure data of all materials in the database. Based on the high-symmetry point path information contained in the data, the band gap values at each high-symmetry point were calculated, and the band gaps at the X, Y, Z and Γ points (${Gap}_{X}$, ${Gap}_{Y}$, ${Gap}_{Z}$ and ${Gap}_{\Gamma }$,) were taken as the primary prediction targets. Prior to model training, we performed data preprocessing to remove anomalous samples and extreme outliers. From a physical perspective, ultra-wide-bandgap materials are rare in practical optoelectronic applications and generally suffer from higher calculation uncertainty in first-principles simulations, and these extreme samples would impair the model’s fitting performance for the mainstream band gap range. Accordingly, we set independent one-sided upper truncation thresholds for the four targets: 6 eV for ${Gap}_{X}$ and ${Gap}_{\Gamma }$, 7 eV for ${Gap}_{Y}$, 8 eV for ${Gap}_{z}$. The statistical distributions of the four curated high-symmetry band gaps are visualized in Figure A3. After preprocessing, the final valid sample sizes for model training and evaluation are 10,146 (${Gap}_{X}$), 10,871 (${Gap}_{Y}$), 15,194 (${Gap}_{Z}$), and 23,163 (${Gap}_{\Gamma }$), respectively. The full workflow of data curation and model evaluation is summarized in Figure A1.

Ablation studies are widely utilized to validate the efficacy of individual input features and quantify their respective contributions to predictive performance. To quantify the physical properties to model performance, we performed ablation studies using the Full Model (FM) as the baseline. Six ablation control groups were established by excluding individual feature categories: density (w/o Den), unit cell volume (w/o Vol), lattice constant a (w/o Lat_a), lattice constant b (w/o Lat_b), lattice type (w/o LT), and space group (w/o SG). To ensure a rigorous control of variables, all ablation configurations maintained the same model architecture, training hyperparameters, dataset splits, and random seeds as the baseline (Table 3). As shown in Table 4, the experimental results show that the Full Model (FM) achieves the lowest MAE across all targets, indicating that all selected features contribute to the model’s performance. Specifically, removing the space group (w/o SG) has the most significant impact on $Gap_{X}$, increasing the MAE from 0.35 to 0.48. For $Gap_{Z}$, omitting the unit cell volume (w/o Vol) leads to the highest error (MAE of 0.58). Other features, such as density and lattice constants, also exhibit varying degrees of importance depending on the specific band gap being predicted. This indicates that integrating these diverse physical and structural properties is beneficial for improving overall prediction accuracy. Physically, the observed trends are consistent with the symmetry-driven origin of electronic energy bands, where symmetry features like space groups and lattice types govern band degeneracy and dispersion relations at high-symmetry points. As these features act as primary structural determinants of the electronic bandgap, their omission inevitably leads to a visible decline in predictive accuracy.

**Figure 4:**
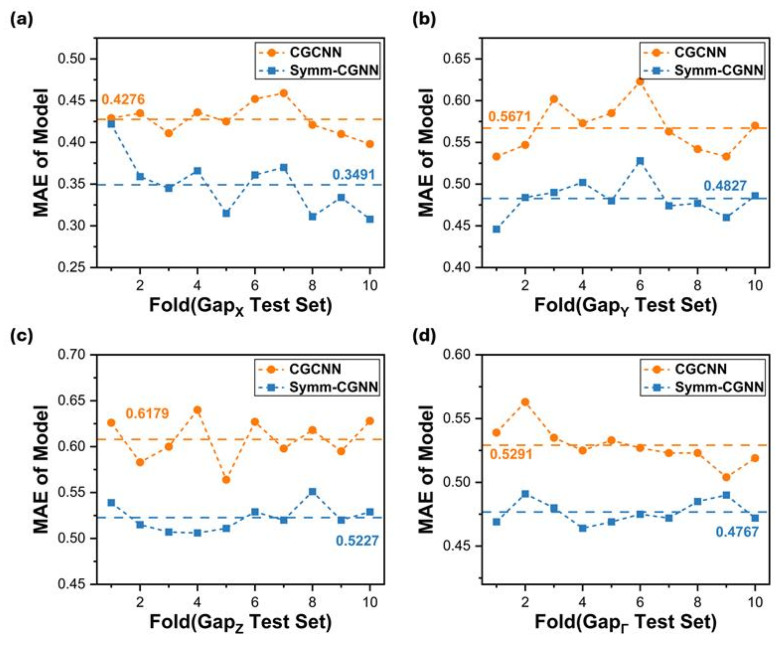
Predictive performance comparison under 10-fold cross-validation. (**a**) MAE for the ${Gap}_{X}$ prediction across 10 independent data folds. (**b**) MAE for ${Gap}_{Y}$ the prediction across 10 independent data folds. (**c**) MAE for the ${Gap}_{Z}$ prediction across 10 independent data folds. (**d**) MAE for the ${Gap}_{\Gamma }$ prediction across 10 independent data folds.

**Figure A4:**
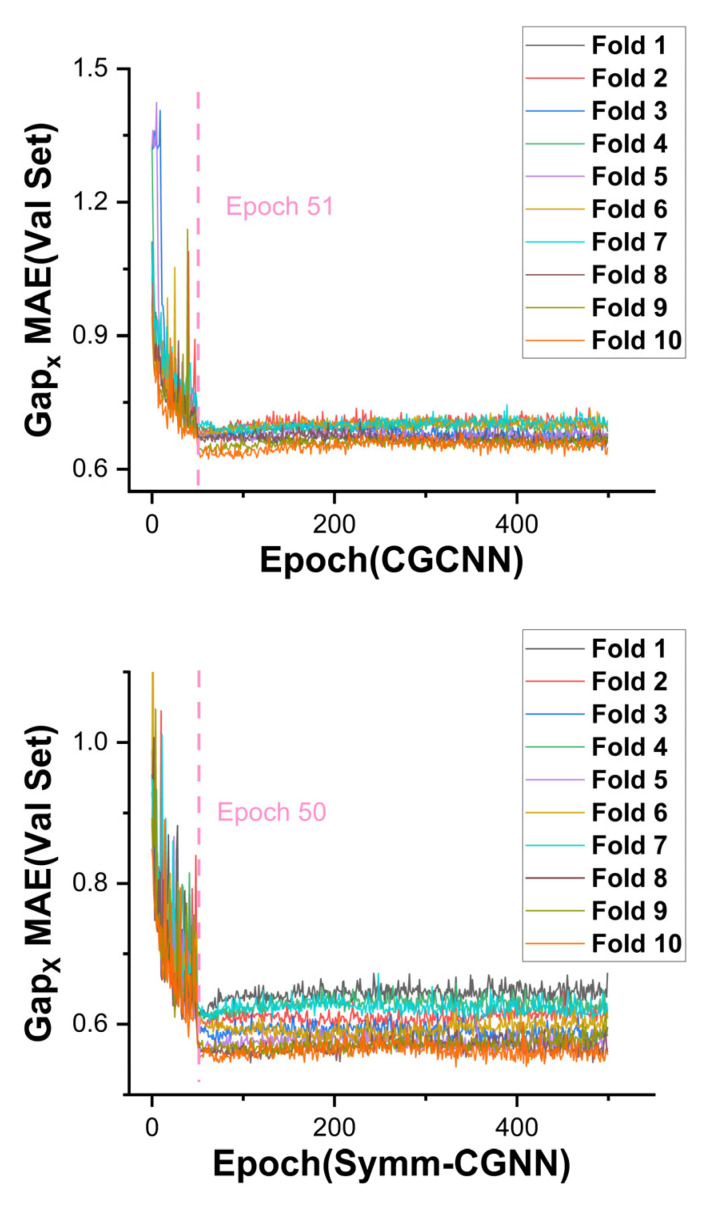
Validation MAE curves over 500 epochs for ${\mathrm{Gap}}_{X}$ predictions. The upper and lower plots display the convergence behaviors of the baseline CGCNN and the proposed Symm-CGNN frameworks during the 10-fold cross-validation, respectively. The distinct colored lines represent the 10 independent folds, while the vertical dashed lines indicate the approximate epochs where the models achieve stable convergence.

**Figure A5:**
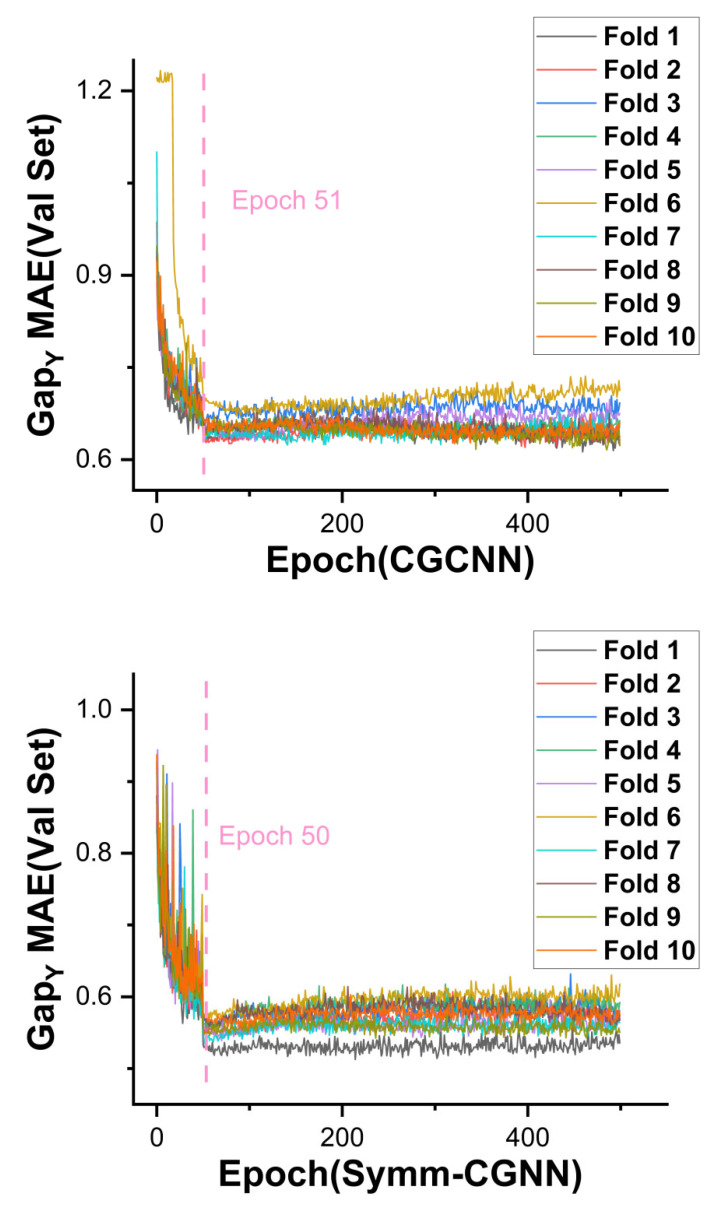
Validation MAE curves over 500 epochs for ${\mathrm{Gap}}_{Y}$ predictions. The upper and lower plots display the convergence behaviors of the baseline CGCNN and the proposed Symm-CGNN frameworks during the 10-fold cross-validation, respectively. The distinct colored lines represent the 10 independent folds, while the vertical dashed lines indicate the approximate epochs where the models achieve stable convergence.

**Figure A6:**
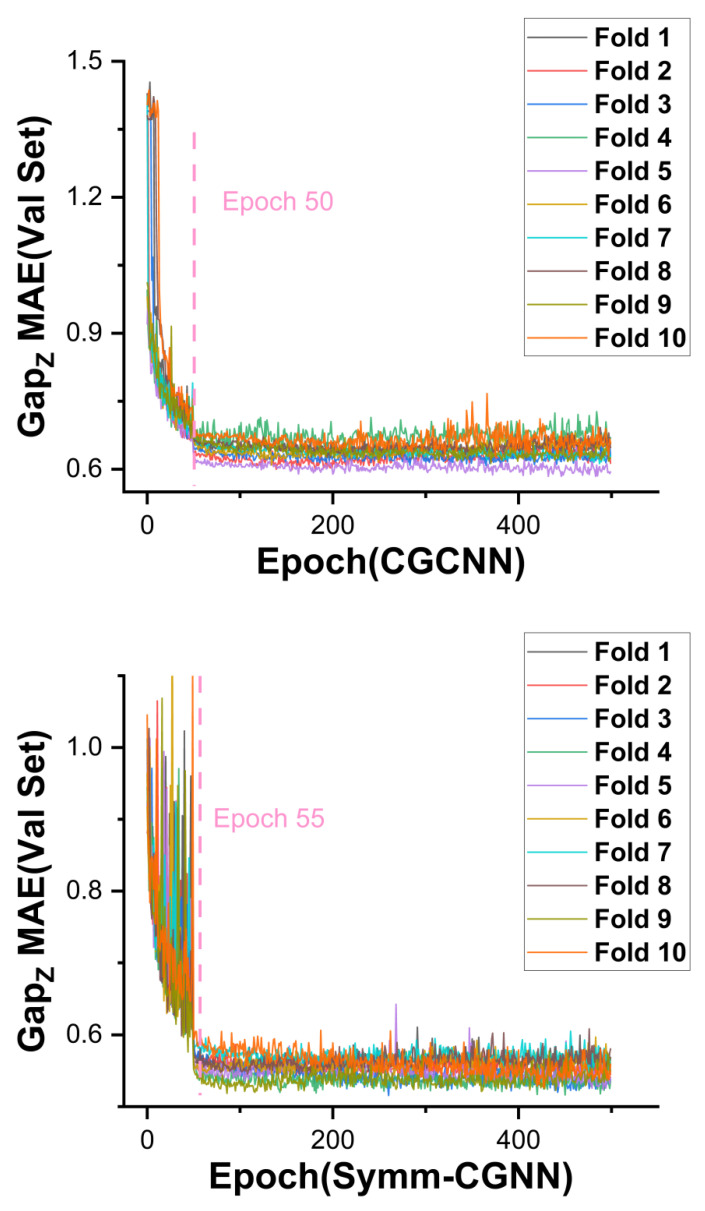
Validation MAE curves over 500 epochs for ${\mathrm{Gap}}_{Z}$ predictions. The upper and lower plots display the convergence behaviors of the baseline CGCNN and the proposed Symm-CGNN frameworks during the 10-fold cross-validation, respectively. The distinct colored lines represent the 10 independent folds, while the vertical dashed lines indicate the approximate epochs where the models achieve stable convergence.

**Figure A7:**
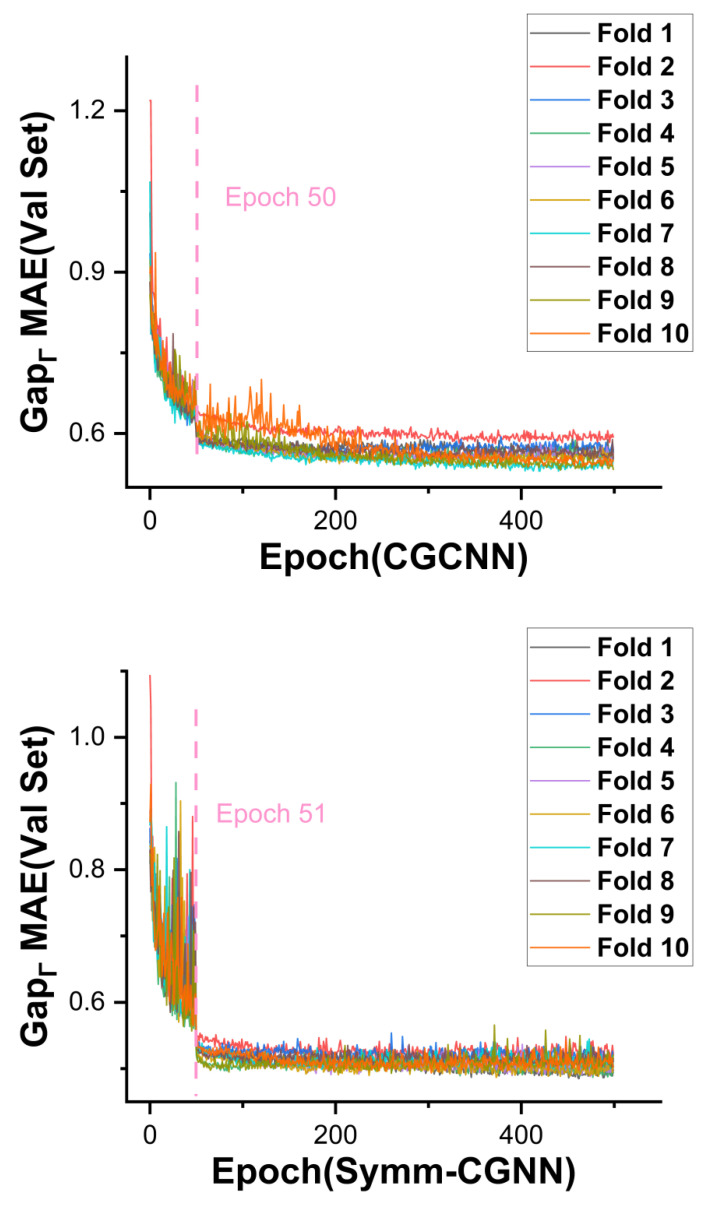
Validation MAE curves over 500 epochs for ${\mathrm{Gap}}_{\Gamma }$ predictions. The upper and lower plots display the convergence behaviors of the baseline CGCNN and the proposed Symm-CGNN frameworks during the 10-fold cross-validation, respectively. The distinct colored lines represent the 10 independent folds, while the vertical dashed lines indicate the approximate epochs where the models achieve stable convergence.

A 10-fold cross-validation was conducted to evaluate the model’s generalization capability. The full validation MAE convergence curves for all four high-symmetry band gaps during cross-validation are provided in Figure A4, Figure A5, Figure A6 and Figure A7. Across the 10 independent folds, the Symm-CGNN framework yields lower Mean Absolute Errors (MAEs) for all high-symmetry points compared to the baseline CGCNN model (Figure 4). Specifically, the 10-fold average MAEs achieved by the Symm-CGNN are 0.3491 (${Gap}_{X}$), 0.4827 (${Gap}_{Y}$), 0.5227 (${Gap}_{Z}$), and 0.4767 (${Gap}_{\Gamma }$), which are lower than the corresponding CGCNN averages of 0.4276, 0.5671, 0.6179, and 0.5291. Furthermore, Symm-CGNN exhibits narrower error variances across the 10 folds, indicating improved prediction stability across different data splits. These results validate our core hypothesis: while conventional local graph convolutions struggle to differentiate complex electronic states, the injection of macroscopic crystal symmetry features (e.g., space groups and lattice parameters) successfully forcibly guides the network. This global-local fusion enables the Symm-CGNN to capture the underlying physical constraints of the Brillouin zone.

The predictive performance of Symm-CGNN was evaluated on independent test sets and compared against three baseline models: CGCNN, MEGNet, and ALIGNN. As shown in Table 5, where the reduction percentages represent the relative decrease in MAE of Symm-CGNN compared to each baseline model, Symm-CGNN consistently achieves lower MAE values across all four target properties. These relative error reductions range from 2% to 31%. The improvement is particularly notable for $Gap_{X}$, where Symm-CGNN reduces the prediction error by more than 25% against MEGNet and ALIGNN. For the other three targets ($Gap_{Y},\;Gap_{Z},\;and\;Gap_{\Gamma }$), the model maintains a steady advantage, with error reductions typically ranging between 7% and 16%. These results indicate that incorporating structural symmetry features improves band gap prediction accuracy across various high-symmetry directions. Specifically, Symm-CGNN yields a more substantial performance gain over CGCNN, which encodes no explicit crystallographic symmetry information. In comparison, the improvement is relatively moderate against MEGNet and ALIGNN, likely because these two models already integrate local geometric descriptors (such as bond angles) that implicitly capture certain symmetry constraints. This trend further underscores the critical role of symmetry information in predicting band gaps at high-symmetry points.

**Figure 5:**
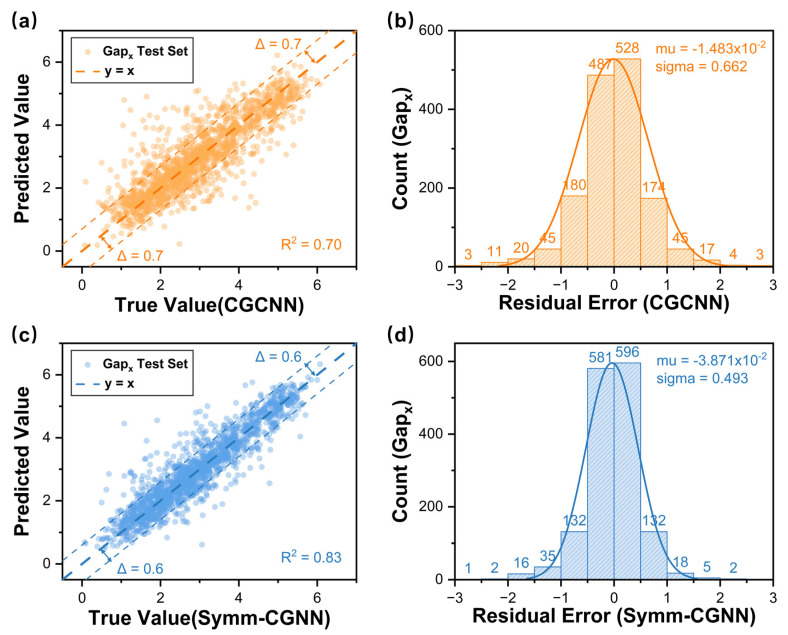
Comparison of prediction accuracy and residual error distribution for ${\mathrm{Gap}}_{X}$ between CGCNN and Symm-CGNN. (**a**,**b**) predicted versus true values and the corresponding residual error distribution for the CGCNN. (**c**,**d**) predicted versus true values and the corresponding residual error distribution for the Symm-CGNN.

To evaluate the model’s predictive performance across all target high-symmetry band gaps, we first analyze $Gap_{X}$ as a representative case. The baseline CGCNN yields an $R^{2}$ of 0.70 with a prediction error envelope of Δ = 0.70 eV (Figure 5a). In comparison, Symm-CGNN achieves an $R^{2}$ of 0.83 and a narrower envelope of Δ = 0.60 eV, showing tighter clustering of the predicted values along the diagonal (Figure 5c). This improvement is also reflected in the residual error distributions. The baseline CGCNN has an error distribution with a mean (μ) of −0.01483 and a standard deviation (σ) of 0.662 (Figure 5b). For Symm-CGNN, the σ decreases to 0.493 (Figure 5d), indicating a narrower error spread and reduced prediction variance.

**Figure 6:**
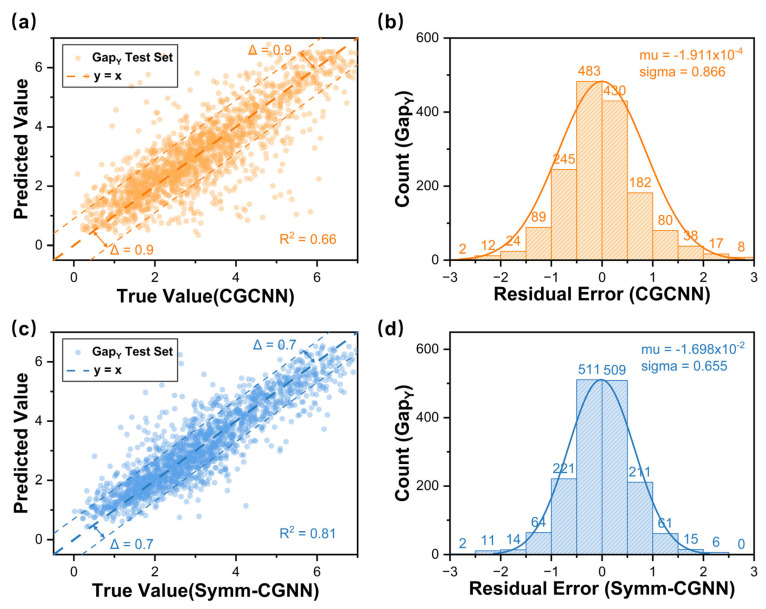
Comparison of prediction accuracy and residual error distribution for ${\mathrm{Gap}}_{Y}$ between CGCNN and Symm-CGNN. (**a**,**b**) predicted versus true values and the corresponding residual error distribution for the CGCNN. (**c**,**d**) predicted versus true values and the corresponding residual error distribution for the Symm-CGNN.

For $Gap_{Y}$, Symm-CGNN demonstrates a consistent improvement. The CGCNN baseline obtains an $R^{2}$ of 0.66 with an error envelope of Δ=0.9 eV (Figure 6a). In contrast, Symm-CGNN increases the $R^{2}$ to 0.81 and narrows the error envelope to Δ=0.7 eV, demonstrating a tighter concentration of predicted values along the diagonal (Figure 6c). Regarding residual distributions, the standard deviation of the prediction errors drops from 0.866 for CGCNN to 0.655 for Symm-CGNN, while the mean residual remains close to zero for both models (Figure 6b,d).

**Figure 7:**
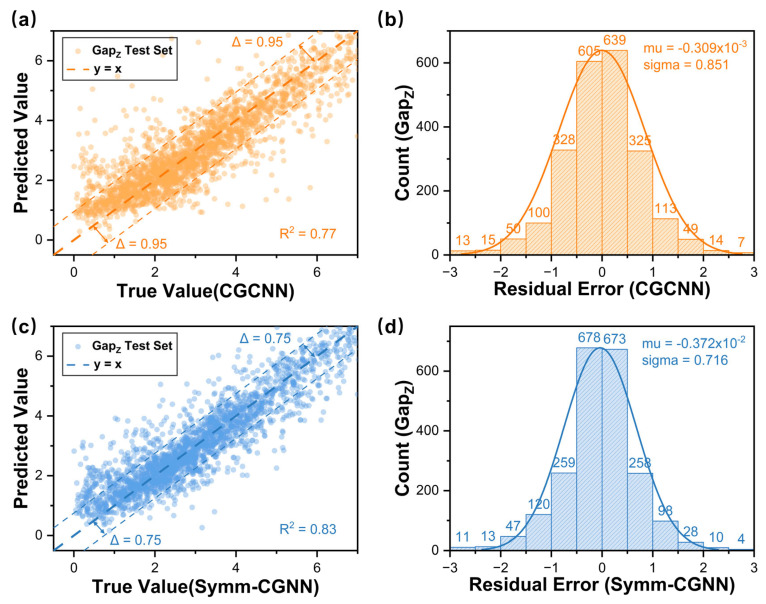
Comparison of prediction accuracy and residual error distribution for ${\mathrm{Gap}}_{Z}$ between CGCNN and Symm-CGNN. (**a**,**b**) predicted versus true values and the corresponding residual error distribution for the CGCNN. (**c**,**d**) predicted versus true values and the corresponding residual error distribution for the Symm-CGNN.

In terms of $Gap_{Z}$, CGCNN yields an $R^{2}$ of 0.77 and an error envelope of Δ=0.95 eV (Figure 7a), whereas Symm-CGNN achieves an $R^{2}$ of 0.83 with a reduced error envelope of Δ=0.75 eV (Figure 7c). The residual standard deviation decreases from 0.851 to 0.716 upon incorporating symmetry information, indicating the model’s enhanced prediction consistency (Figure 7b,d).

**Figure 8:**
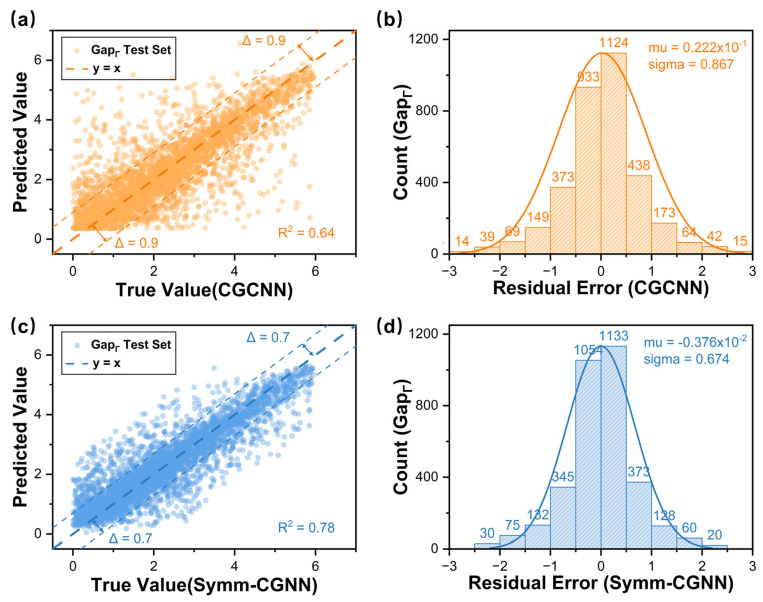
Comparison of prediction accuracy and residual error distribution for ${\mathrm{Gap}}_{\Gamma }$ between CGCNN and Symm-CGNN: (**a**,**b**) predicted versus true values and the corresponding residual error distribution for the CGCNN; (**c**,**d**) predicted versus true values and the corresponding residual error distribution for the Symm-CGNN.

For $Gap_{\Gamma }$, which possesses the largest sample size, CGCNN yields and $R^{2}$ of 0.64 with an error envelope of Δ=0.7 eV (Figure 8a). Symm-CGNN improves the $R^{2}$ to 0.78 and narrows the error envelope (Figure 8c). The standard deviation of the residual distribution falls from 0.867 to 0.674, indicating that incorporating global symmetry features consistently reduces prediction variance across the evaluated high-symmetry points (Figure 8b,d).

From the MAE results, it can be observed that the Symm-CGNN consistently outperforms the CGCNN across different prediction targets. This indicates a better fit to the data and lower prediction errors, demonstrating a significant overall improvement in predictive capability.

## 4. Discussion

Overall, our Symm-CGNN model delivers remarkably favorable prediction performance for band gaps at high-symmetry points, outperforming conventional local graph neural network baselines across all evaluation metrics. Unlike conventional local-graph models constrained by short-range atomic environments, Symm-CGNN incorporates macroscopic parameters—such as volume, density, lattice constants, and space groups—to enhance prediction accuracy for symmetry-dependent band gaps at high-symmetry points. We attribute this performance improvement to the physical nature of reciprocal space, where electronic energy states at specific k-points are governed by the crystal’s long-range periodicity and global symmetry operations (such as space groups) that cannot be fully characterized by local chemical environments alone. Crystals with similar local atomic arrangements can form distinct macroscopic lattices; consequently, without global symmetry information, purely local models may fail to distinguish these structural differences, leading to deviations in reciprocal-space property predictions. The introduced space groups and lattice constants provide Symm-CGNN with essential geometric boundary conditions and physical constraints, supporting more reliable predictions of band gaps at high-symmetry points.

Building on the effectiveness of this symmetry-integration strategy, the model’s performance could be further enhanced in future work. For instance, applying this framework to models that already incorporate local geometric descriptors (such as bond and dihedral angles in ALIGNN), or adopting the design principles of equivariant neural networks, represents a promising path toward higher accuracy [45,46]. Additionally, expanding the training dataset scale in the context of high-throughput materials research is expected to further improve the model’s prediction accuracy and generalization capability.

## 5. Conclusions

This study addresses the challenge of predicting band gaps at high-symmetry points in crystalline materials. To overcome this issue, we built the Symm-CGNN model based on the CGCNN framework, which incorporates the global geometric and symmetry features of crystals and encodes them into high-dimensional representations. The architecture combines local structural awareness with global geometric constraints. Evaluated on a dataset combining the MP and 2DMat databases, Symm-CGNN achieves a 26% reduction in prediction MAE relative to the CGCNN baseline. In summary, Symm-CGNN improves the predictive accuracy of high-symmetry band gaps, addressing the limitations of conventional models in distinguishing band gaps across the Brillouin zone. By reducing prediction errors in both 3D and 2D material tasks, this model provides a tool for predicting electronic structure properties and offers a methodological framework for subsequent studies on band-edge renormalization, photoexcited transitions, and optoelectronic properties.

## Figures and Tables

**Table 1:** Elemental properties used for atomic node feature representation.

Property	Unit	Range	Categories
Group number	-	1, 2,…, 18	18
Period number	-	1, 2,…, 9a	9
Electronegativity	-	0.5–4.0	10
Covalent radius	pm	25–250	10
Valence electrons	-	1, 2,…, 12	12
First ionization energy	eV	1.3–3.3	10
Electron affinity	eV	−3–3.7	10
Block	-	s, p, d, f	4
Atomic volume	cm^3^/mol	1.5–4.3	10

**Table 2:** The macroscopic structural properties used in Global features.

Property	Unit	Range	Categories
Lattice a	Å	2–18	11
Lattice b	Å	2–18	11
Volume	${Å}^{3}$	0–5000	26
Density	$g/cm^{3}$	0–3.6	11
Lattice type	-	0, 1,…, 6	7
Space group	-	0, 1,…, 230	16

**Table 3:** Hyperparameter configurations for the neural network model.

Category	Hyperparameter	Value
Architecture	Number of convolutional layers	3
	Number of hidden layers	3
Training	Optimizer	Adam
	Learning rate	0.01
	Batch size	64
	Epochs	500
Regularization	Dropout fraction	0.1
	Weight decay	1 × 10^−4^

**Table 4:** The MAE comparison of different ablation groups on bandgap predictions.

Group	Ablation Study MAE Comparison			
	$Gap_{X}$	$Gap_{Y}$	$Gap_{Z}$	$Gap_{\Gamma }$
FM	0.35	0.48	0.52	0.48
w/o Den	0.44	0.51	0.55	0.53
w/o Lat_a	0.36	0.53	0.56	0.51
w/o Lat_b	0.37	0.51	0.56	0.52
w/o LT	0.36	0.50	0.57	0.50
w/o Vol	0.44	0.52	0.58	0.52
w/o SG	0.48	0.51	0.57	0.52

**Table 5:** The table summarizes the MAE of Symm-CGNN and three baseline models. The reduction percentage represents the relative MAE decrease of Symm-CGNN with respect to each corresponding baseline model.

Target	Symm-CGNN	CGCNN		MEGNet		ALIGNN	
		MAE	Reduction	MAE	Reduction	MAE	Reduction
${\mathrm{Gap}}_{X}(\mathrm{eV})$	0.35 ± 0.03	0.43	18%	0.51	31%	0.48	27%
${\mathrm{Gap}}_{Y}(\mathrm{eV})$	0.48 ± 0.02	0.57	16%	0.55	13%	0.52	8%
${\mathrm{Gap}}_{Z}$(eV)	0.52 ± 0.01	0.62	16%	0.59	12%	0.56	7%
${\mathrm{Gap}}_{\Gamma }(\mathrm{eV})$	0.48 ± 0.01	0.53	9%	0.56	14%	0.49	2%

## Data Availability

The datasets used in this study were obtained from the Materials Project and 2DMatpedia.

## Abbreviations

The following abbreviations are used in this manuscript:

Symm-CGNN	Symmetry-Information-Enhanced Crystal Graph Neural Network
CGCNN	Crystal Graph Convolutional Neural Network
MAE	Mean Absolute Error
${Gap}_{X}$	The electronic band gap at the X high-symmetry point
${Gap}_{Y}$	The electronic band gap at the Y high-symmetry point
${Gap}_{Z}$	The electronic band gap at the Z high-symmetry point
${Gap}_{\Gamma }$	The electronic band gap at the Γ high-symmetry point
